# The future of affordable cancer immunotherapy

**DOI:** 10.3389/fimmu.2023.1248867

**Published:** 2023-09-06

**Authors:** Niels Schaft, Jan Dörrie, Gerold Schuler, Beatrice Schuler-Thurner, Husam Sallam, Shiri Klein, Galit Eisenberg, Shoshana Frankenburg, Michal Lotem, Areej Khatib

**Affiliations:** ^1^ Department of Dermatology, Friedrich-Alexander-Universität Erlangen-Nürnberg, Universitätsklinikum Erlangen, Erlangen, Germany; ^2^ Comprehensive Cancer Center Erlangen European Metropolitan Area of Nuremberg (CCC ER-EMN), Erlangen, Germany; ^3^ Deutsches Zentrum Immuntherapie (DZI), Erlangen, Germany; ^4^ Bavarian Cancer Research Center (BZKF), Erlangen, Germany; ^5^ Molecular Genetics and Genetic Toxicology, Health Science Department, American Arab University, Ramallah, Palestine; ^6^ Sharett Institute of Oncology, Hadassah Hebrew University Hospital, Jerusalem, Israel; ^7^ Hadassah Cancer Research Institute, Hadassah Hebrew University Hospital, Jerusalem, Israel; ^8^ Women's Health Research Unit, The Research Institute of the McGill University Health Centre, Montreal, QC, Canada

**Keywords:** immunotherapy, affordable, adoptive cell therapy, microbiome, RNA-based vaccines, biomarkers, immunohistochemistry

## Abstract

The treatment of cancer was revolutionized within the last two decades by utilizing the mechanism of the immune system against malignant tissue in so-called cancer immunotherapy. Two main developments boosted cancer immunotherapy: 1) the use of checkpoint inhibitors, which are characterized by a relatively high response rate mainly in solid tumors; however, at the cost of serious side effects, and 2) the use of chimeric antigen receptor (CAR)-T cells, which were shown to be very efficient in the treatment of hematologic malignancies, but failed to show high clinical effectiveness in solid tumors until now. In addition, active immunization against individual tumors is emerging, and the first products have reached clinical approval. These new treatment options are very cost-intensive and are not financially compensated by health insurance in many countries. Hence, strategies must be developed to make cancer immunotherapy affordable and to improve the cost-benefit ratio. In this review, we discuss the following strategies: 1) to leverage the antigenicity of “cold tumors” with affordable reagents, 2) to use microbiome-based products as markers or therapeutics, 3) to apply measures that make adoptive cell therapy (ACT) cheaper, e.g., the use of off-the-shelf products, 4) to use immunotherapies that offer cheaper platforms, such as RNA- or peptide-based vaccines and vaccines that use shared or common antigens instead of highly personal antigens, 5) to use a small set of predictive biomarkers instead of the “sequence everything” approach, and 6) to explore affordable immunohistochemistry markers that may direct individual therapies.

## 1 Introduction

Immunotherapy has changed the cancer treatment scenario and revolutionized tumor immunology. Immunotherapy treatments, such as adoptive T-cell therapy (ACT) or the use of immune checkpoint inhibitors (ICIs), are now well-established components of the toolbox of cancer treatments, significantly improving longevity in a substantial proportion of patients (1–3). The vast amount of ongoing research in the field is expected to enhance the essential role of immunotherapy in cancer treatment.

However, with the advancing success of cancer immunotherapy, it is becoming clear that a significant drawback of current immunotherapies is their high expense. To enable the wide usage of immunotherapy, efforts will eventually have to be centered on developing immunotherapy treatments that are significantly cheaper and affordable to larger populations worldwide.

Getting a cancer immunotherapy treatment costs more than a house in many cities in the US and is more expensive than putting a few children through private college. The average cost of cancer drugs increased from $50,000 per patient in the mid-1990s to $250,000. That is four times the median US household annual income. Immunotherapies often cost more than $100,000 per patient. For some of the newest immunotherapies, the price tag is even steeper: When including the value of the medical support necessary to deliver these treatments, a price tag of $850,000 per patient is not unheard of (4). For example, although the wholesale acquisition cost of CAR-T-cell therapies to treat B-cell lymphoma is $373,000, a new study by Prime Therapeutics of real-world data found that the total cost averages more than $700,000 and can exceed $1 million in some cases (5).

Increasingly, approaches to treat solid tumors and hematological malignancies involve the concurrent administration of several products with distinct but complementary mechanisms of action in combination or close sequence as part of a regimen that also seeks to minimize the development of drug resistance (6–8). The use of combined immunotherapies means that costs can quickly double or triple. Some recent examples include the addition of pertuzumab to trastuzumab for the treatment of human epidermal growth factor receptor-2 (HER-2)-positive breast cancer and the use of programmed cell death protein (PD-1) and programmed cell death ligand (PD-L1) inhibitors in combination with anti-cytotoxic T-lymphocyte-associated protein 4 (CTLA-4) therapies in metastatic melanoma. This trend presents serious challenges for Health Technology Assessment (HTA) bodies and payers. Combination regimens are expected to increase over the next few years (7, 9). Almost all information regarding the costs of immunotherapy is based on data from OECD countries; however, access to oncology medicines remains unequal across OECD/EU countries. The charges in non-OECD countries will probably be higher and may enjoy less support from health or insurance institutions or drug companies. Additionally, there is little doubt that the population of third-world countries will mostly be unable to cope with such expenses.

**Figure 1:**
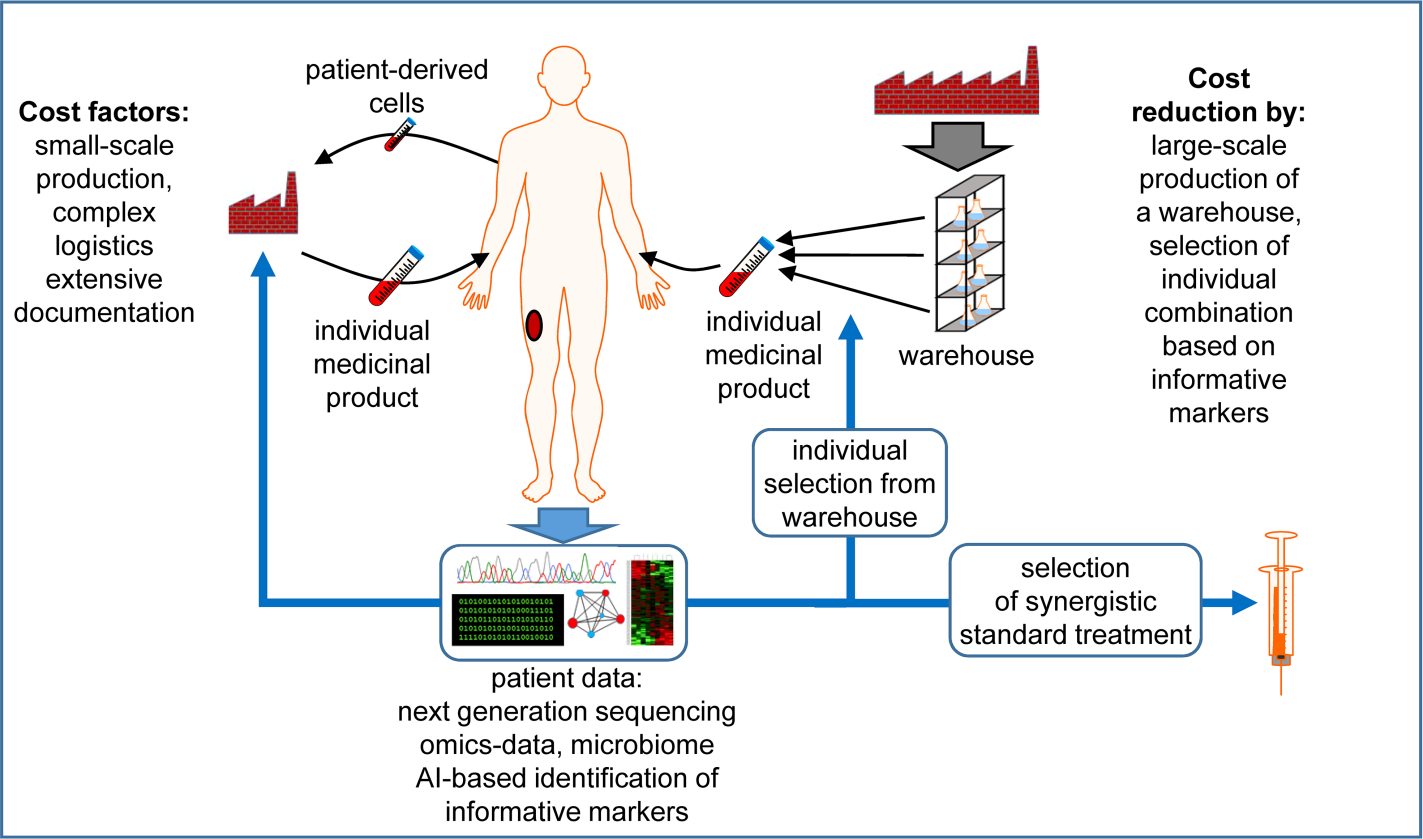
Factors that contribute to the high costs of individualized medicinal products and possibilities for cost reduction. The production of cellular therapeutics usually takes place on a per-patient basis, i.e. each patient requires a personal small-scale production of the individualized medicinal product in a specialized facility under labor-intensive documentation. Source materials are usually patient-derived living cells, which increases the logistic effort. Next generation sequencing and other omics data are exploited to define individual antigens, which are synthesized in a personalized manner. Alternatively, therapeutic components could be produced at larger scale, increasing the economic efficiency, creating a warehouse of constituents. Using individual patient data, possibly exploited with the help of Artificial Intelligence (AI) to identify a manageable set of informative markers, an individual combination of these elements is selected to generate the individualized product. When possible, truly individual components are avoided or reduced to a minimum, including patient-derived cells. To improve the efficiency of the treatment further, the in depth data analysis can propose the use of established thus cheaper drugs in combination with the advanced individualized medicinal products. See the main text for further details. The Motifolio Scientific Illustration Toolkit was used for the generation of this figure.

The future of cancer immunotherapy will largely depend on the ability of researchers to make it affordable to larger populations. This review summarizes some scientific suggestions for making this happen (**Figure 1**).

## 2 Leverage the antigenicity of “cold tumors” with affordable reagents

One of the most consistent predictors for the success of immune checkpoint inhibitors (ICI) in metastatic patients is the general load of missense mutations and the density of lymphocytic infiltrate in the tumors (10–12). The accepted paradigm for the contribution of non-synonymous mutations or frameshifts is that they generate altered peptide epitopes that work as neo-antigens (13–15). Unlike wild-type sequences, these neo-antigens have not induced a tolerizing mechanism. Consequently, T-cell clones can emerge, which recognize these neo-epitopes with high affinity and effectively destroy cancer cells (16, 17). The power of neo-antigen-cognate T cells in the clinic was shown in several pioneering works by Rosenberg et al., targeting four mutant proteins in a patient with breast cancer (NCT01174121), and by Tran et al., targeting mutant KRAS G12 (18–20).

The situation is very different in cancers referred to as “cold tumors” or “immune deserts,” two descriptions relating to the scarcity of immune targets and effector T cells. Among these are uveal melanoma, pancreatic cancer, ovarian and breast cancers, and any cancer with loss of HLA class I, mutations in β2 microglobulin, and defects in antigen presentation (21–24).

Neo-epitopes are not solely generated by mutations. In the absence of genomic-encoded antigens, the mRNA transcript, or the actual protein product itself, is sought as a source for immunogenic neo-epitopes. The concept that defects in any of the ribosomal proteins (DRiPs) will yield impaired peptides and enrich the immune-peptidome to be detected by the immune system was described by Yewdell et al. but was not leveraged towards a therapy (25). Admon et al. described that, following viral infection, large numbers of HLA class I peptides derive from DRiPs (26). Thus, it was proposed that damaging ribosomal proteins will enhance the anti-viral immune response; this may also apply to cancers (27).

A renewed interest in this approach was evoked by Abdel-Wahab and colleagues, showing that in blood malignancies with mutant splicing factors, novel splicing-derived proteins may appear (28). Similarly, Oka et al. show in lung cancer cell lines that ablations of the nonsense-mediated mRNA decay (NMD) factor UPF1, and a splicing factor, SF3B1, are found to increase the proportion of aberrant transcripts (29). Taking one further step forwards, Lu et al. used a pharmacological compound, indisulam, which enhances the degradation of the RNA-binding motif protein 39 (RBM39), which often is upregulated in cancers (30). Indisulam and other sulfonamides can affect splicing in tumor cells at a concentration that may be safe to use in the clinic. Most intriguingly is the demonstration that true neo-epitopes emerged by clinical-grade pharmaceutics, primarily due to intron retention.

In summary, DriPs and peptide products of splice-disrupted mRNA can be induced in cancer cells. This especially applies to cancers harboring oncogenic splicing factor mutations, which have limited benefit from ICI: acute myeloid leukemias, uveal melanoma, myelodysplastic syndrome, and non-small-cell lung cancer. Rapid screens of small molecule libraries and antitumor antibiotics are highly encouraged. If issues of patenting and IP are put aside, these compounds may be cheap to produce and replace the expensive cell therapies that are among the few options for these “cold” tumors.

## 3 Microbiome-based products

There is growing evidence that gut microbiota is related to immunotherapy outcomes. For example, it has been shown that transcriptionally expressed metagenomic pathways in the gut microbiome are related to progression-free survival in melanoma (31). Results from a study by Nomura et al. suggest that fecal short-chain fatty acid (SCFA) concentrations may be associated with PD-1 inhibitor efficacy; thus, SCFAs may be the link between the gut microbiota and PD-1 inhibitor treatment outcome. Because fecal examinations are entirely non-invasive, they may be applicable for routine monitoring of patients (32). Recently, a correlation between gut bacterial composition and prognosis in hepatocellular carcinoma patients suggested a potential role for the gut microbiome as a prognostic marker for the response to nivolumab (33) and the response to anti-CD19 CAR-T-cell therapy in patients with B-cell malignancies (34). Another study demonstrated that secondary resistance and immune-related adverse events are related to longitudinal dynamics of the intestinal microbiota in patients with advanced malignancies (35).

That the gut microbiome can affect the immune response was already shown by Gur et al. in 2015. They found that a bacterium from the oral cavity directly interacted with TIGIT to diminish NK- and T-cell functionality (36). Since then, an emerging body of evidence has implicated host-intrinsic microorganisms in influencing the response to cancer immunotherapy (37). Attempts to translate microbiome-based therapies, e.g., in melanoma patients, have mild success (NCT03353402) (38, 39). Still, the gut microbiota diversity in individuals of different ethnicities and geographic areas makes it difficult to standardize therapeutic formulations. Despite these problems, techniques of fecal transplantation will remain cheap and accessible and are currently being tested in several clinical trials (NCT05502913 (40), NCT05286294, NCT04975217). The potential synergy between gut bacteria and ICI will not only increase the response rate but may shorten the time to achieve these benefits, which is also tested in many clinical trials, e.g., in liver cancer (NCT05750030, NCT05690048) (41), lung cancer (NCT05669846, NCT04924374), colorectal cancer (NCT05279677, NCT04729322) (42), melanoma (NCT05251389 (43), NCT04988841, NCT04577729, NCT03341143) (44), kidney cancer (NCT04758507) (45), gastrointestinal cancer (NCT04130763) (46), prostate cancer (NCT04116775), and mesothelioma (NCT04056026) (47).

## 4 Can adoptive cell therapy be made affordable?

Cell therapy consists of cellular “drugs” prepared mostly in local production facilities. The long manufacturing time, complex delivery systems, and discrete and per-patient production are only some of the hurdles affecting the time-to-market and manufacturing costs of cell-based therapeutics.

Most cell therapies developed in recent years, approved and in clinical pipelines, use autologous cell products. The personalized generation of cellular products tailored to fit a specific antigen or disease condition has advanced immensely, with feasible applications. Although autologous cells benefit from the advantage of avoiding rejection, using allogeneic cells offers scalable production from abundant cell sources. Therefore, significantly simplifying and expediting manufacturing turns the product more affordable and thus allows many more patients to be treated. Albeit these advantages, allogeneic cells trigger graft versus host disease (GvHD) or vice versa- host versus grafted lymphocytes, due to HLA mismatched α/β T cells.

Using allogeneic cell sources that elicit minimal immunogenic reactions is one approach for reducing GvHD. NK cells are one of the options for this type of cell source. Pioneering work from the Ruggeri group shows that KIR-mismatched alloreactive donor NK cells protected patients from AML relapse with no GvHD (48). NK cells also do not produce IL-1 and IL-6, the main cytokines involved in cytokine release syndrome (CRS), minimizing one of the main adverse events of current cell therapy (49). Allogeneic NK CD19 chimeric antigen receptor (CAR) cells derived from cord blood have a 73% response rate without significant toxic effects in lymphoma and chronic lymphatic leukemia (CLL) patients (50). Many ongoing clinical trials use CAR-NK targeting various antigens, including CD19 (e.g., NCT05487651, NCT05410041), EGFR, EpCAM, GD2, mesothelin (NCT03692637), and HSP70 (51).

The ability of iNKT cells to rapidly respond to lipid antigens and secrete a wide variety of cytokines has placed these cells at the frontlines of many types of immune responses (52), including cytotoxic responses, which can lead to tumor lysis, recruitment of other innate- and adaptive-related immune cells, and regulation of immunosuppression (53). These responses, robust in mouse models and humans, are problematic in cancer patients since their number in the peripheral blood of these patients is significantly decreased (54–56). In addition, their functionality is hampered in these patients, as shown by their lower secretion of IFNγ and a tendency to a Th2 phenotype. These facts make their potential application for human immunotherapy problematic (52, 54).

Alternatively, γδ T cells can be used as an allogenic source since they do not recognize MHC molecules and are hence not alloreactive (57–59). It was shown that γδ T cells – retrovirally transduced or RNA-transfected with an αβ TCR against, e.g., cytomegalovirus (CMV) or a tumor antigen – were highly functional *in vitro* (60, 61) and in mice (62, 63). Also, CARs were functionally introduced into γδ T cells (61) and are even tested in clinical trials (NCT04107142, NCT04735471 (64), NCT05302037). An additional advantage of CAR-transfected γδ T cells is that they produce lower quantities of cytokines compared to CAR-transfected αβ T cells, reducing the risks of CRS (61).

Recently, a population of unconventional innate-like T cells, mucosal-associated invariant (MAIT) cells, has elicited hopes for efficient off-the-shelf, allogeneic immunotherapy for two main reasons. First, their semi-invariant αβ T-cell receptor recognizes small-molecule biosynthetic derivatives of riboflavin synthesis presented on the restriction molecule major histocompatibility complex (MHC)-related protein-1 (MR1). As a result, MAIT cells do not recognize autoantigens or induce graft-versus-host disease (GvHD). Second, MAIT cells are strong cytotoxic cells that secrete pro-inflammatory cytokines and lyse infected cells using granzyme B and perforin. Taken together, these characteristics justify the efforts and enthusiasm that are being invested in this population to achieve a new approach to immunotherapy (65).

Mesenchymal stem cells (MSCs) are also considered a source of evasive immune cells. They are highly immunosuppressive, diminishing T-cell activation and antigen-presenting cell maturation and, in this way, they delay allo-rejection (66). However, since MSCs have been used to deliver cytotoxic reagents into tumors with limited efficacy (67), further studies are needed to exploit their therapeutic potential.

A different approach for generating universal cell sources exploits the vast advances in cell engineering, turning allogeneic cellular products into less immunogenic ones. Genome editing using CRISPR-Cas9 or similar editing systems targeting the β2-microglobulin HLA class I molecule and the T-cell receptor (TCR) in combination with CAR expression has been used to create universal CAR-T cells that are less prone to attack autologous T cells (68). These combined efforts reduce GvHD but also host versus graft (HvG), allowing for a broader therapeutic window for CAR-T cells. The CAR construct is often introduced into the TRAC, TRBC1, or TRBC2 locus to create TCR knockout cells and regulate the CAR expression through the TCR promoter (69). A retrospective comparison between auto-CD19 CARs and allo-CD19 CARs showed only minor GvHD in allo-CARs. Nonetheless, the response rate was favorable toward the allo-CAR with 100% at nine months follow-up compared to 88% in the auto-CAR. This advantage was attributed to the combined signals in allo-CAR of TCR and CAR (70). Clinical testing of allogeneic CAR-T trials directed at hematological and solid tumors is ongoing in many centers. Targets for these CAR-T trials include CD19, BCMA (71), and CD20 in hematological tumors, and GD2, mesothelin (NCT03545815), CD70 (NCT05795595, NCT04438083, NCT04696731), MUC1-C [NCT05239143 (72)], and NKG2DL in solid tumors.

Another option for producing off-the-shelf cell products is performing genetic editing on induced pluripotent stem cells (iPSCs) before cellular differentiation. Following manipulation, these cells can be differentiated into many types, including T cells, NK cells, and dendritic cells. Allo-iPSCs can be used from either a matched homogenous genetic background individual or following allele-specific editing. These cells can be manipulated to avoid GvHD and HvG by HLA pseudo-homozygosity, escaping recognition by both T and NK cells (73). Further manipulations, such as the expression of CD47 and HLA-G, can mediate escape from NK and macrophages, creating ‘universal’ iPSCs (74). Clinical trials using NK derived from iPSCs were completed or are ongoing in solid tumors and hematological malignancies (NCT03841110, NCT04630769, NCT05182073).

Hematopoietic stem cells (HSCs) possess unlimited expansion capacity and can differentiate into multiple cell types. Conventional sources of HSCs include adult bone marrow and the umbilical cord of newborns. An additional method to achieve a high number of HSCs uses iPS cells, which have high scalability due to the robustness of their cell culture conditions. HSCs derived from cord blood or bone marrow are currently being evaluated to manufacture CAR-HSCs, which can differentiate into effector cells, including CAR-T and CAR-NK cells. Interestingly, stem cell-derived T cells have a unique cytokine profile with fewer safety risks (75, 76).

The production costs of TCR-T or CAR-T cells can be reduced by transfection of mRNA encoding the receptors into T cells instead of using viral transduction for receptor transfer. In addition to being an easier process than viral transduction, receptor-RNA transfection of T cells (or any other cells) can even be performed decentralized with, e.g., closed electroporation systems, making local and cheaper production possible (77). Another advantage is that CAR-RNA transfection has a favorable toxicity profile considering possible on-target/off-tumor reactions due to its transient effects. Two clinical studies showed that on-target/off-tumor toxicity could cause severe problems and even death if the receptor is introduced by stable viral transduction (78, 79). By transient transfection of T cells, the receptor expression is temporarily restricted, rendering potential off-target and on-target/off-tumor toxicity also transient. The CAR-RNA transfer strategy is especially attractive in preclinical and phase I clinical trials exploring new tumor antigens for CAR-T-cell therapy with an unknown clinical safety profile. The mRNA transfection strategy for CARs was proposed by us some time ago (80) and has, in the meantime, been applied in several clinical trials in patients with breast cancer and melanoma (NCT01837602 NCT03060356; targeting c-MET) (81) and mesothelioma, pancreatic cancer, and ovarian cancer (NCT03608618, NCT01897415, NCT01355965 targeting mesothelin) (82–84). RNA transfection was even explored with non-solid tumors using CD19 and CD123 as target antigens (NCT02277522, NCT02624258, NCT02623582) (85). The mRNA-CAR-T cells in these studies were well tolerated, migrated to primary and metastatic tumor sites, showed clinical antitumor activity, and showed no evidence of on-target/off-tumor toxicity against normal tissues (81, 82). However, the transient receptor expression *per se* necessitates repetitive injections. Unlike virally transduced cells, which have to be given only once and proliferate in the patient’s body, RNA-transfected cells will lose CAR expression and must be replenished to maintain cytolytic pressure on the tumor. This might, in turn, increase the treatment costs if many more cells need to be produced.

The significant number of approaches being actively evaluated to make adoptive cell therapy affordable, only some of which are described here, point toward the high expectations of the scientific community and overall raise hopes for widespread immunotherapy, which may be shortly more than a wishful dream.

## 5 RNA-based therapeutic cancer vaccines

In the past decade, RNA therapeutics have witnessed a true revolution. Several RNA-based therapies have been approved by the FDA for treating genetic diseases, with unprecedented success, as in spinal muscular atrophy (86–88). Moreover, recent years showed the world that RNA-based therapies, specifically mRNA vaccines, can be the answer to a pandemic and save the lives of millions.

However, in the field of cancer treatments, RNA therapies are lagging. The rapidly adaptable mRNA vaccines against Covid-19 ended years of concerns regarding the large-scale feasibility of RNA-based therapeutics. In addition to a vast amount of clinical data on safety and efficacy, pharmaceutical companies augmented their production capabilities, and new solutions to incurable diseases, mainly cancer, can now be sought.

However, due to its high antigen heterogeneity, cancer represents a significant challenge in the design of therapeutic cancer vaccines. RNA-based cancer vaccines can encode individually mutated neo-antigens, resulting in their presentation, which is a very personalized medicinal product, and, therefore, very cost intensive. Finding these mutations involves high costs for the sequencing of the tumor, usually also involving challenging logistics and centralized sequencing facilities. A possibility to reduce this expense may be the use of new decentralized 3^rd^-generation sequencing technologies which offer much better cost efficiency. Very recently, Moderna and Merck announced that mRNA-4157/V940, an investigational personalized mRNA cancer vaccine, in combination with Keytruda^®^ (Pembrolizumab), was approved as a breakthrough therapy by the FDA for adjuvant treatment of patients with high-risk melanoma following complete resection (NCT03897881) (89, 90). Several other clinical trials, both in the adjuvant and metastatic setting, are running (e.g., NCT04161755 in pancreatic cancer (91), NCT02316457 in triple-negative breast cancer, NCT03815058 in melanoma, NCT04486378 in colorectal cancer, NCT03480152 in gastrointestinal cancer (92), NCT05761717 in hepatocellular carcinoma, and NCT03289962 in several solid tumors).

Alternatively, an off-the-shelf approach can also be chosen if the vaccines are based on prepared mRNAs encoding non-mutated antigens often expressed in the tumor, reducing costs. Examples of this exist for ovarian carcinoma treated with a liposome-formulated mRNA vaccine encoding three ovarian carcinoma tumor-associated antigens (TAA) (NCT04163094), melanoma treated with a liposome-formulated mRNA vaccine encoding four selected malignant melanoma-associated antigens: New York-ESO 1 (NY-ESO-1), tyrosinase, melanoma-associated antigen A3 (MAGE-A3), and trans-membrane phosphatase with tensin homology (TPTE)(NCT02410733) (93), prostate cancer (NCT04382898 (targeting five different antigens), NCT00831467), and non-small cell lung cancer (NCT05142189, NCT03164772 (with six target antigens), NCT00923312 (with five target antigens). However, a “one size fits all”-tumor vaccine formulation does not exist. Since individual tumors from even a narrowly defined cancer type still vary substantially in their antigen expression even at different sites, any pre-selection of defined antigens will always be a compromise between comprehensiveness and cost efficiency. Here, an individually defined cocktail prepared from an off-the-shelf tumor antigen warehouse could be a more cost-efficient solution (94). Although this requires determining the individual tumor’s antigen expression again, decentral field sequencing technologies like the Oxford Nanopore™ platform could offer a cheaper option.

Adding an adjuvant is beneficial to achieve an effective immune response against a cancer vaccine antigen. Several approaches are followed to reduce costs for such adjuvants. For example, one can re-purpose effective immune adjuvants with no intellectual property (e.g., Freund’s, BCG, Alum). Moreover, one can also combine the RNA-based vaccines described above with a fraction of double-stranded (ds)RNA resulting in an adjuvant-like stimulus through NFκB activation by Toll-like receptor 3 (TLR3), which binds to the dsRNA (95), or by complexing a fraction of the mRNA with protamine, which then acts as an adjuvant that induces an effective immune response through TLR7-mediated signaling (96, 97).

Over the last 20 years, a well-established approach was to transfect dendritic cells (DCs) with mRNA *ex-vivo* and inject those cells to induce antitumor immune responses. Although a slow but constant improvement concerning immunologic activity was achieved during this period, this technology never made it to a broader clinical application. The *ex-vivo* production of such an individualized cellular product never met a sufficient cost-effectivity ratio to be commercially attractive. The only DC-based cancer vaccine that received clinical approval was sipuleucel-T (Provenge™) produced by Dendreon Corporation, which consisted of a DC-enriched PBMC fraction pulsed with a GM-CSF/PAP fusion molecule and was discontinued for commercial reasons (98, 99). Performed under the required high standards of good manufacturing practice (GMP), the production costs to treat one patient are within the range of ten thousands of dollars without any revenues. Retail prices would be significantly higher if a customary profit margin was intended. Nevertheless, the highly controlled surrounding of the large number of trials provided a cornucopia of valuable information and insights translated into vaccination approaches (100, 101), in which the antigen was given to target APCs *in vivo* for expression of the antigen (102). The rapid implementation of mRNA-based vaccines against Covid-19 would not have been possible without all the existing data generated in the field of mRNA-based tumor vaccination both with *ex-vivo* transfected DCs and via the application of mRNA-based formulations *in vivo*.

The following hurdles must be tackled to facilitate affordable mRNA-based cancer vaccines: 1) Tumor sequencing must be fast and cheap to allow a tailored individual selection of antigens from a pre-produced warehouse of mRNAs, possibly via panel sequencing on decentralized field sequencing devices. 2) RNA production must be economical. The production of large batches of mRNA can achieve this. However, producing individual mRNAs for only one patient will not be feasible. 3) RNA must be formulated to be stable at -20°C to circumvent excessively complex transport and storage logistics. 4) The other components of the vaccine formulation must be affordable. 5) The additional expenses for GMP compliance must be limited. While safety must be maintained, bureaucracy must be reduced.

The last section focused on RNA-based cancer vaccines, although there are other formats in which antigens may be provided. Specific epitopes can be delivered as synthetic peptides (101, 103), and whole tumor antigens as full-length proteins. Even complete tumor cells can be lysed and used as antigen source. All these approaches have been tested in humans, while peptides appear to be the most promising competitor of RNA (103). Although we focused on RNA-based strategies in the section above, all limitations, lines of reasoning, and rationales discussed more or less apply to the latter approaches of cancer vaccination as well.

## 6 A small set of predictive biomarkers instead of the “sequence everything” approach

Currently, the most applied approach in cancer immunotherapy is targeting immune checkpoints or immune regulatory molecules, which have shown high success rates in several clinical trials. Melanoma is a highly mutated cancer with a wide frequency range, of 0.1-100 somatic mutations per Megabase (MB). In a study on 3083 matched tumor-normal pairs from 27 different tumor types, melanoma was found to have the highest mutational frequency of all cancers analyzed (104). Two studies that performed whole-exome sequencing on tumor samples of melanoma patients showed improved clinical outcomes after being treated with checkpoint inhibitors in patients with a high mutational burden (105, 106). Therefore, whole-exome sequencing is being used by some groups to identify mutational load as a biomarker to give patients the advantage of immunotherapy.

On the other hand, studies using smaller gene panels (170-500 genes) have shown that the total exomic mutational burden can be extrapolated, and, more important, also the response to immunotherapy can be predicted. In a study with 65 melanoma patients, the mutational burden calculated using FoundationOne (315 genes) was found to be significantly associated with treatment response and survival, particularly at >20 mutations/MB) (107). Therefore, determining mutational load using smaller panels may also be a biomarker of response to immunotherapy with much lower costs. An additional benefit of such sequencing panels may lie in a better selection of therapeutic alternatives besides immunotherapy. Regulatory pathology and oncology bodies such as the College of American Pathologists (CAP) have adopted this minimalistic approach and recommend a panel of BRAF, NRAS, and KIT mutations as a routine in melanoma patients (108). The identification of mutations in tumor samples of melanoma patients can even be customized in simple multiplex PCR assays for labs with limited resources. Our group has tested tumor samples of a small cohort of cutaneous melanoma patients using the Trusight Oncology 500 panel. The analysis showed that all samples had a high mutational burden, ranging from ~5-48 mutations per MB. All samples were found to have one or more mutations in BRAF, NRAS, and/or KIT that could be used in targeted therapy.

In conclusion, genomic tests on tumor samples can be run with a small, cost-effective panel to identify the mutational burden and to allow decisions regarding treatment with targeted therapy and immunotherapy.

## 7 Exploring affordable immunohistochemistry markers that may direct individual therapies

Traditionally, immunohistochemistry (IHC) is used as a tool to help the pathologist confirm the cancer diagnosis. Thus the method is routinely established in oncologic centers worldwide, and the required equipment is available. While targeted therapies and immune checkpoint inhibitors have demonstrated remarkable efficacy, these drugs do not show uniform responses in all patients. Immunohistochemistry has emerged as a promising tool for assessing the expression of specific proteins within tumor samples that may predict response. Among those proteins, programmed death ligand 1 (PD-L1), T-cell markers, and mitotic index markers are used the most.

Immunohistochemical analysis of PD-L1 expression in melanoma samples has shown correlations with response to immune checkpoint inhibitors such as Pembrolizumab (Keytruda) and Nivolumab (Opdivo) (109). High PD-L1 expression is associated with improved response rates and increased overall survival in some studies, suggesting PD-L1 as a potential predictive biomarker (110).

Furthermore, tumor-infiltrating lymphocytes (TILs) within the tumor microenvironment have been linked to better treatment outcomes in cancer, especially melanoma (111). Objective assessment of TILs has traditionally been performed by flow cytometry to derive T-cell lineage (112). However, immunohistochemistry can also quantify TIL subsets, including CD8^+^ cytotoxic T cells and CD4^+^ helper T cells (113). In addition, IHC staining of FoxP3 can help evaluate the presence and density of Tregs within the tumor microenvironment (114).

Many studies tried to connect one or more other IHC stainings with prognosis and response to therapy, such as mitotic index and angiogenesis markers (115). Additionally, BAP1 (BRCA1-associated protein 1) and MITF (microphthalmia-associated transcription factor) expression were linked to poor prognosis (116, 117).

Thus, using IHC of a limited set of markers can be a cost-efficient tool to direct clinical treatment decisions.

## 8 Conclusions

Cancer is a common disease that affects many humans. New technologies helped to understand the molecular basis of the different malignancies and their interplay with the human immune system. They led to new treatment strategies, some turning a previously fatal diagnosis into a treatable and even curable condition. However, in many cases, this comes with a price tag of several hundred thousand dollars. Even in developed countries, this is a financial burden that is hard to bear for society and unbearable for most individuals. Hence, economic considerations are crucial for the general use of the new drugs. The biggest cost drivers are, on the one hand, the high grade of personalization, often involving the individual production of cellular products, and on the other hand the successive administration of the different advanced medicinal products, due to the nescience, which product is clinically effective. The first could be addressed by an individualized combination of components from a warehouse of products, thus allowing a more economic production. The use of *in-vivo*-targeted substances like mRNA can help to reduce or avoid the cost-intensive employment of living cells. The second could be tackled by implementing new kinds of patient data, while narrowing the information from established technologies to an informative set of markers, which aid in treatment selection, thus avoiding the trial and error principle. In addition, supportive therapies, which are *per se* inexpensive, but increase the response rate to the advanced treatments can decrease overall costs. Hopefully, the ideas and proposals mentioned above will raise awareness of this dilemma and contribute to developing cost-efficient and clinically effective treatment strategies.

## Author contributions

NS, JD, SF, and ML contributed to the conception and design of the review. NS, JD, SK, SF, ML, and AK wrote the first draft of the manuscript. All authors contributed to the article and approved the submitted version.
